# Pedunculated Extraluminal Gastrointestinal Stromal Tumor of Stomach: A Rare Case Report in South Korea

**DOI:** 10.1155/cris/7884178

**Published:** 2026-06-09

**Authors:** Moonjin Kim

**Affiliations:** ^1^ Department of Surgery, Incheon St. Mary’s Hospital, College of Medicine, The Catholic University of Korea, Incheon, Republic of Korea, catholic.ac.kr

## Abstract

Gastrointestinal stromal tumors (GISTs) are known to arise from any of the five layers of the gastric wall, most commonly from the muscularis propria. Extraluminal gastric GISTs account for approximately 8% of cases and are relatively rare, with pedunculated forms being scarcely reported in South Korea. A 25‐year‐old man presented to our hospital with an incidental left upper quadrant (LUQ) mass adjacent to the stomach on a screening computed tomography (CT) scan. He had no symptoms and no suspicious findings on gastrointestinal endoscopy. Laparoscopic excision was performed, revealing an 8 cm × 7 cm mass connected to the posterior gastric serosa. Immunohistochemical staining confirmed the diagnosis of GIST. We report this case as an extremely rare presentation of a gastric GIST presenting as a pedunculated mass connected to the outer serosal surface, underscoring the diagnostic challenges associated with such atypical presentations.

## 1. Introduction

Gastrointestinal stromal tumors (GISTs) are rare tumors that originate in the mesenchyme of the gastrointestinal tract [1]. These tumors are found primarily in adults, with the most common site of occurrence being the stomach. Gastric GISTs are believed to be caused by the abnormal growth of Cajal cells, KIT‐positive spindle cells around the myenteric plexus [2]. These cells are essential for regulating the motility of the gastrointestinal tract. GISTs are characterized by the expression of cell surface markers such as CD117 (KIT) and CD34. The majority of GISTs are known to be driven by activating mutations in the c‐kit proto‐oncogene, resulting in the autonomous activation of the tyrosine kinase receptor KIT. Additionally, some GISTs may arise from activating mutations in the PDGFRA gene [3]. Radiologically, most GISTs appear as solid masses on abdominal computed tomography (CT) scans or MRI, often exhibiting central necrosis with focal or multifocal hemorrhage. They are typically seen attached to the submucosa of the gastric wall on endoscopic examination. Endoscopic biopsy is often avoided due to the potential risk of tumor cell dissemination and laparoscopic/open excision may be the only way to make histological diagnosis [1]. For that reason, definitive diagnosis prior to histopathologic confirmation is challenging for gastric GIST. In addition, there are other types of mesenchymal tumors in gastrointestinal tract that can partially overlap with GIST [4].

We recently encountered a case of an intra‐abdominal mass adjacent to the stomach identified on abdominal CT. The esophagogastroduodenoscopy (EGD) showed normal gastric wall findings, so we considered sarcoma or neurofibroma as the presumptive diagnosis before surgery, but the surgical findings and postoperative histology were diagnosed as pedunculated exophytic or serosa originated gastric GIST. Extraluminal gastric GISTs have an incidence of approximately 8% and are relatively rare, and there were two case reports similar to the present case [4, 5]. We would like to announce this report as a very rare gastric GIST case that occurred in South Korea.

## 2. Case Presentation

A 25‐year‐old man visited our hospital after a CT scan, performed during a routine health checkup, revealed a 7.2 cm × 4.4 cm mass in the left upper quadrant (LUQ) (Figure 1). Physical examination and laboratory results were unremarkable.

**Figure 1:**
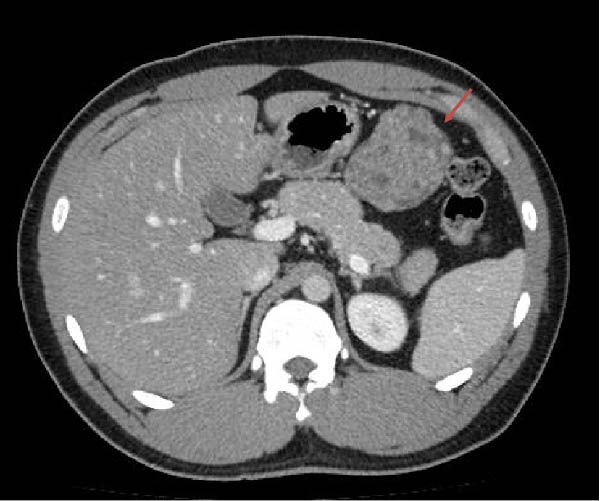
Abdomen contrast CT. A 7.2 cm × 4.4 cm sized heterogeneousely enhancing mass is noted in LUQ (red arrow). It is adjacent to gastric body, greater curvature side.

An EGD performed at the screening center showed no abnormalities in the gastric wall (Figure 2). Although the mass was located adjacent to the greater curvature of the gastric body on CT, the entire gastric wall, including the submucosa, appeared structurally intact—an atypical finding for gastric GIST.

**Figure 2:**
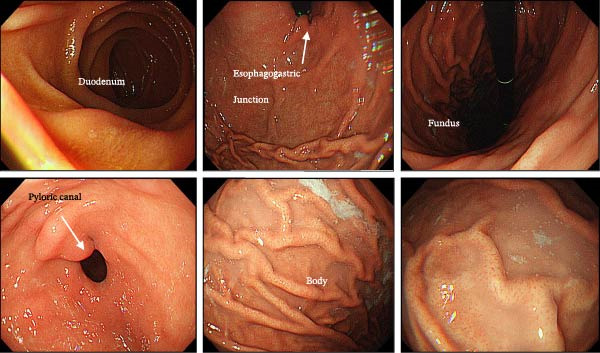
View of esophagogastroduodenoscopy. No lesion was seen.

Laparoscopic resection was planned. Intraoperatively, a large mass was found tethered to the gastric serosa via a fibrous cord. The tumor, along with the connecting cord and adjacent serosal tissue, was completely resected using Harmonic shears (ETHICON). The serosal defect was repaired with 3–0 Monofix sutures (Figure 3). The operation was completed without any intraoperative complications.

**Figure 3:**
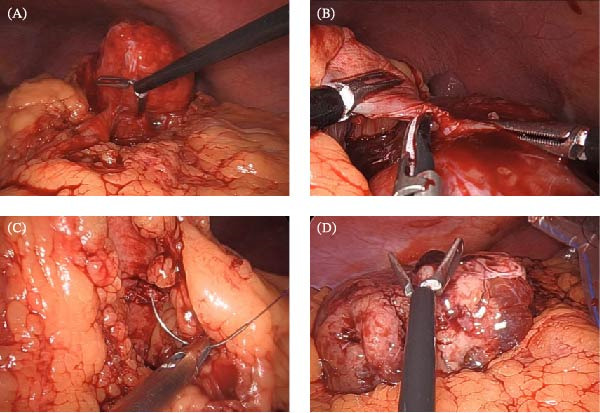
Operative view of patient. (A) After exposure of greater sac, the mass was found adjusted to the greater curvature of stomach. (B) The mass was linked to gastric serosa. Dissection and ligation of adhesion between mass and gastric serosa was done by harmonic device. (C) For the complete excision of mass, ligation was done closed to stomach, and serosa defect closure was performed by monofix 3–0. (D) Final description of mass. The mass was extracted using laparoscopic bag.

The gross appearance of the surgical specimen is shown in Figure 4. As observed intraoperatively, the specimen included a portion of the gastric serosa, and pathological examination confirmed clear resection margins, indicating successful en bloc resection. Histopathological examination confirmed the diagnosis of GIST. Hematoxylin and eosin (H&E) staining demonstrated spindle cell morphology typical of GIST. Immunohistochemical staining showed positivity for CD34, CD117, actin, and DOG‐1, which is specific for GIST. Negative staining for S‐100 and desmin helped exclude other differential diagnoses such as schwannoma, leiomyoma, and sarcoma (Figures 5, 6) [6].

**Figure 4:**
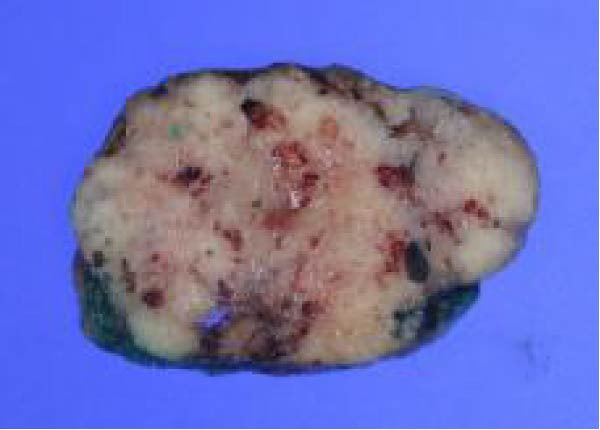
The gross section of surgical specimen.

**Figure 5:**
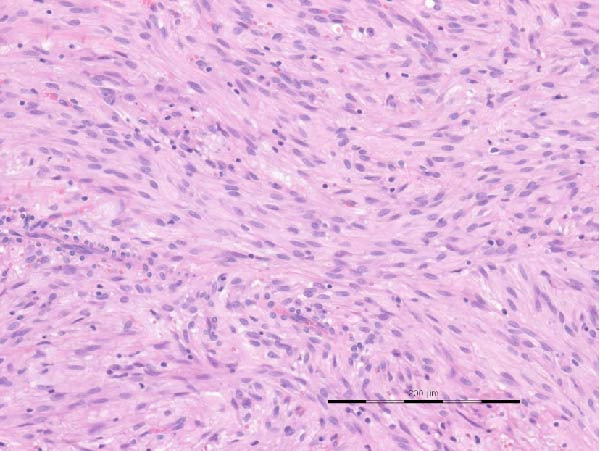
Hematoxylin and eosin staining of specimen. Spindle cells are shown (×200).

**Figure 6:**
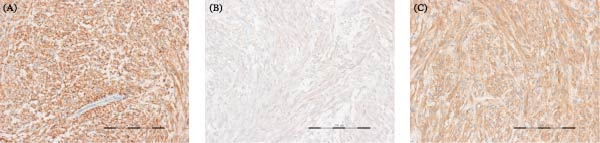
Immunohistochemical staining (×200). (A) Positive staining of CD34. (B) Positive staining of CD117 (c‐kit). (C) Positive staining of DOG‐1.

The tumor measured 8 cm × 7 cm × 4.5 cm and exhibited a low mitotic rate (3 per 50 high‐power fields), low cellularity, and negative resection margins. According to the NIH consensus criteria it was classified as intermediate risk, while under the Armed Forces Institute of Pathology (AFIP) criteria it was considered low risk [7]. Therefore no adjuvant treatment was administered, and the patient was managed with regular surveillance. Follow‐up abdomen and chest CT was performed at 3‐month intervals after surgery, and at the current 1‐year mark, no recurrence has been observed.

## 3. Discussion

This case highlights the discrepancy between the preoperative differential diagnosis based on imaging studies, including CT and EGD, and the actual diagnosis determined by intraoperative findings and pathological examination. Preoperatively, retroperitoneal schwannoma or sarcoma was considered the most likely diagnosis. However, during surgery, the mass was found to be isolated from adjacent organs and was connected to the gastric serosa by an adhesive band‐like structure. Final pathological examination confirmed the diagnosis of gastric GIST. As in the present case, it can be difficult to differentiate GISTs from schwannoma or sarcoma based on initial imaging studies such as CT. However, immunohistochemical staining showed negativity for S‐100 and positivity for CD117, DOG‐1, and CD34, effectively ruling out schwannoma.

This form of extraluminal gastric GIST appears to be extremely rare, and to date, very few similar cases have been reported in South Korea. Since GISTs typically originate from interstitial cells of Cajal (ICCs)—which are CD34‐positive and located within the gastrointestinal wall—the serosal or subserosal origin represents a very uncommon variant [4]. Another possibility is that exophytic gastric GISTs are relatively common, and from a pathological perspective, the mass in this case may have originated from the muscular layer and grown outward, rather than arising from the serosa. However, the exact layer of origin of the mass could not be definitively identified on pathological examination. Cases of pedunculated exophytic masses connected to the gastric serosa by a band‐like structure, as in this case, are extremely rare, with only one case reported in South Korea [5]. This rarity complicates preoperative diagnosis, making intraoperative evaluation critical. Therefore, in cases involving ambiguous intra‐abdominal masses, prompt surgical intervention for pathological confirmation should be prioritized over extended preoperative workup.

A limitation of this case report is that Ki‐67 proliferation index and molecular testing were not performed, making precise prognostic assessment difficult. However, mitotic count remains the primary factor for prognostic and risk assessment in GIST, and Ki‐67 testing is generally performed selectively. Further case reports are needed to enable a broader understanding of these uncommon forms of gastric GIST.

In conclusion, we report a rare case of a gastric stromal tumor pedunculated form from the serosa, which has been scarcely documented in South Korea. This case may contribute to a better understanding and further research of GISTs. It also highlights the diagnostic challenges of tumors arising from rare anatomical sites, emphasizing the importance of prompt surgical exploration and histopathological confirmation.

## Funding

There are no sources of funding in this article.

## Conflicts of Interest

The author declares no conflicts of interest.

## Data Availability

Data sharing is not applicable to this article as no datasets were generated or analyzed during the current study.
